# Hematocrit, iron and HDL-cholesterol explain 90% of variation in native blood T1

**DOI:** 10.1186/1532-429X-18-S1-O86

**Published:** 2016-01-27

**Authors:** Stefania Rosmini, Heerajnarain Bulluck, Thomas A Treibel, Anish N Bhuva, Amna Abdel-Gadir, Veronica Culotta, Ahmed Merghani, Viviana Maestrini, Anna S Herrey, Charlotte Manisty, James Moon

**Affiliations:** 1 Cardiac Imaging, Barts Heart Centre, London, UK; 2 grid.4464.20000000121612573Department of Cardiovascular Sciences, St Georges, University of London, London, UK; 3 grid.7841.aDepartment of Cardiovascular, Respiratory, Nephrology, Anesthesiology, and Geriatric Sciences, "Sapienza" University of Rome, Rome, Italy

## Background

Native myocardial T1 is known to be affected by variables such as age, gender, heart rate and partial voluming from blood pool. Blood T1 itself has a wide (wider) variability. We aimed to investigate causes of blood T1 variability.

## Methods

77 healthy volunteers with no known cardiovascular condition underwent CMR at 1.5T (Siemens, Avanto). Mid ventricular short axis native T1 maps by MOLLI (with T1* reconstruction in addition) and ShMOLLI were acquired. Hematocrit (Hct), iron profile and lipid profile were acquired immediately prior to the scan. CVI42 (Calgary, Canada) was used for analysis of the maps. A ROI was drawn in the blood pool on the MOLLI T1 map, avoiding papillary muscles and was copied on to the MOLLI T1* and ShMOLLI T1.

## Results

**Figure 1:**
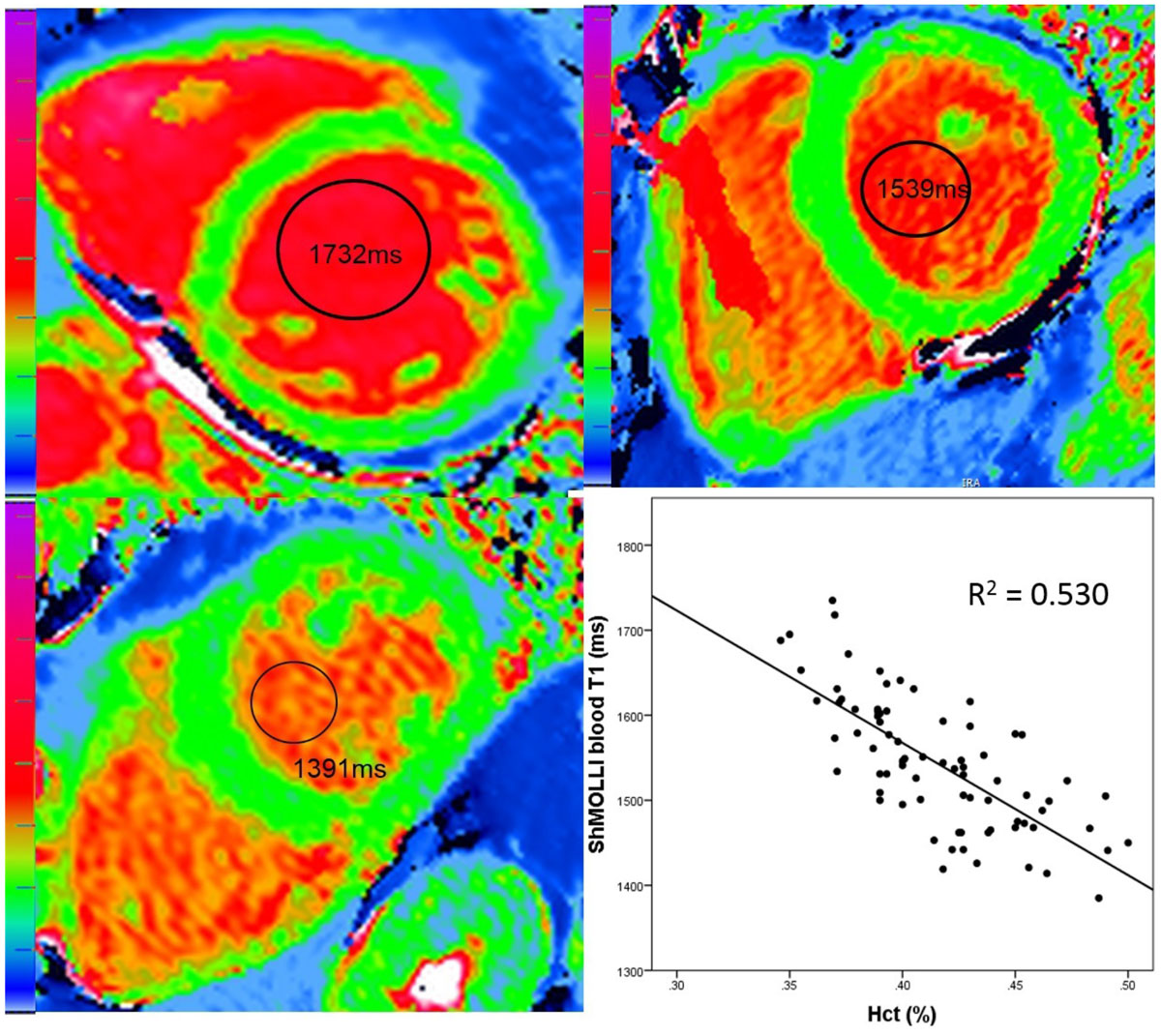
**ShMOLLI T1 maps from 3 healthy volunteers with (top left, top right, bottom right) high, normal and low blood T1**. Comparing high T1 case with low, in this case, both the Hct (37% vs 49%) and the iron were lower (13.2 μmol/L vs 32 μmol/L where normal is 6.6-26 μmol). Bottom right: example correlation, here Blood T1 by ShMOLLI and Hct (R^2^ 0.53, p < 0.0001).

Complete datasets of blood and maps were available for all 77 volunteers (mean age 49 ± 14, range 20-76, 49% males). Mean ± SD of blood T1 by MOLLI T1 was 1638 ± 78 ms, MOLLI T1* 1686 ± 111 ms and ShMOLLI T1 1543 ± 77 ms. There was a negative correlation between blood T1 and Hct (R^2^ 0.530, coeff. -0.728, p < 0.0001)(Figure 1). Hct, iron, HDL-cholesterol, ferritin, triglycerides (TG), LDL-cholesterol and total iron binding capacity (TIBC) resulted to be significant at univariate analysis while this was not the case for albumin and total cholesterol. The multivariate analysis performed including only the significant variables showed that Hct, iron and HDL-cholesterol are significantly correlated with blood T1 by MOLLI T1 and T1* and ShMOLLI (Table 1).

**Table 1:** Univariate and multivariate analysis for blood variables and blood T1 by MOLLI T1, MOLLI T1* and ShMOLLI.

	MOLLI Blood T1			MOLLI Blood T1*			ShMOLLI Blood T1		
UNIVARIATE	R	slope	p	R	Slope	p	R	Slope	p
Hct	-0.672	-1458	< 0.0001	-0.707	-2184	< 0.0001	-0.728	-1556	< 0.0001
Iron	-0.694	-326	< 0.0001	-0.583	-390	< 0.0001	-0.636	-295	< 0.0001
HDL-chol	-0.452	248	< 0.0001	0.427	334	< 0.0001	0.478	260	< 0.0001
Ferritin	-0.309	-61	0.006	-0.367	-103	0.001	-0.336	-65	0.003
TG	-0.321	-109.1	0.004	-0.251	-121.5	0.028	-0.331	-110.7	0.003
LDL-chol	-0.217	-143.1	0.059	-0.122	-114.8	0.291	-0.208	-135.6	0.069
TIBC	0.152	191.2	0.188	0.273	490.1	0.016	0.188	233.8	0.102
Albumin	0.042	1.2	0.717	-0/009	-0.36	0.94	0.023	0.651	0.844
Total cholesterol	0.021	1.8	0.857	0.111	13.5	0.335	0.043	3.6	0.713
**MULTIVARIATE**	**Cum R2**	**Slope**	**p**	**Cum R**	**Slope**	**p**	**Cum R2**	**Slope**	**p**
Hct		-936.5	< 0.0001		-1603.5	< 0.0001		-1085.5	< 0.0001
Iron		-255.1	< 0.0001		-275.5	< 0.0001		-213.9	< 0.0001
HDL-chol	0.88	129.5	< 0.0001	0.831	152.2	0.007	0.884	132.1	< 0.0001

## Conclusions

In health, Hct then iron then HDL-cholesterol explain almost all (90%) of blood T1 variability with anaemia and low iron increasing T1 but with HDL reducing it.

